# Surface Passivation for Promotes Bi-Excitonic Amplified Spontaneous Emission in CsPb(Br/Cl)_3_ Perovskite at Room Temperature

**DOI:** 10.3390/polym15091978

**Published:** 2023-04-22

**Authors:** Saif M. H. Qaid, Hamid M. Ghaithan, Huda S. Bawazir, Abdullah S. Aldwayyan

**Affiliations:** 1 Department of Physics & Astronomy, College of Sciences, King Saud University, P.O. Box 2455, Riyadh 11451, Saudi Arabia; hghaithan@ksu.edu.sa (H.M.G.); 438204457@student.ksu.edu.sa (H.S.B.); dwayyan@ksu.edu.sa (A.S.A.); 2 K. A. CARE Energy Research and Innovation Center, King Saud University, Riyadh 11451, Saudi Arabia; 3 King Abdullah Institute for Nanotechnology, King Saud University, Riyadh 11451, Saudi Arabia

**Keywords:** perovskite, PMMA, thermal evaporation, surface passivation, amplified spontaneous emission, excitons, bi-excitons

## Abstract

Perovskite-type lead halides exhibit promising performances in optoelectronic applications, for which lasers are one of the most promising applications. Although the bulk structure has some advantages, perovskite has additional advantages at the nanoscale owing to its high crystallinity given by a lower trap density. Although the nanoscale can produce efficient light emission, its comparatively poor chemical and colloidal stability limits further development of devices based on this material. Nevertheless, bulk perovskites are promising as optical amplifiers. There has been some developmental progress in the study of optical response and amplified spontaneous emission (ASE) as a benchmark for perovskite bulk phase laser applications. Therefore, to achieve high photoluminescence quantum yields (PLQYs) and large optical gains, material development is essential. One of the aspects in which these goals can be achieved is the incorporation of a bulk structure of high-quality crystallization films based on inorganic perovskite, such as cesium lead halide (CsPb(Br/Cl)_3_), in polymethyl methacrylate (PMMA) polymer and encapsulation with the optimal thickness of the polymer to achieve complete surface coverage, prevent degradation, surface states, and surface defects, and suppress emission at depth. Sequential evaporation of the perovskite precursors using a single-source thermal evaporation technique (TET) effectively deposited two layers. The PL and ASEs of the bare and modified films with a thickness of 400 nm PMMA were demonstrated. The encapsulation layer maintained the quantum yield of the perovskite layer in the air for more than two years while providing added optical gain compared to the bare film. Under a picosecond pulse laser, the PL wavelength of single excitons and ASE wavelength associated with the stimulated decay of bi-excitons were achieved. The two ASE bands were highly correlated and competed with each other; they were classified as exciton and bi-exciton recombination, respectively. According to the ASE results, bi-exciton emission could be observed in an ultrastable CsPb(Br/Cl)_3_ film modified by PMMA with a very low excitation energy density of 110 µJ/cm^2^. Compared with the bare film, the ASE threshold was lowered by approximately 5%. A bi-exciton has a binding energy (26.78 meV) smaller than the binding energy of the exciton (70.20 meV).

## 1. Introduction

The perovskite structures (APbX_3_, where A = MA, FA, and Cs; X = Cl, Br, and I single or mixed variants of binary and ternary halides) are widely used with monovalent A cations and X anions in organic-inorganic hybrid lead halide and inorganic lead halide. Although the potential applications of inorganic cation perovskite materials are mostly focused on light-emitting devices (LEDs), a few reports have shown that these materials have great promise for use in solar cells and photodetectors [1,2]. All of this is particularly attributed to the band gap reliability, short exciton radiation lifetime, and excellent emission with a narrow linewidth (FWHM) and high PLQYs [1,3,4,5,6,7]. The bandgap of perovskite can be tuned according to compositional change using a binary halide ion, such as Cl/Br and Br/I [8,9,10]. Although, theory predicts that the production of binary halide ions from a (Cl/I) is more difficult to combine with mixed halide ions than binary from (Br/Cl) (or) (Br/I) mixture halide ions because of the energies of formation of the halide alloys [8,10]. Looking at the cation part, we can compare the organic with the counterparts from the inorganic hybrids and conclude that CsPbX_3_ has a higher stability level than MAPbX_3_ and FAPbX_3_. Nonetheless, due to the low energies of crystal lattices formations combined with the significant delocalization activity of the surface ions in real operations, CsPbX_3_ remains sensitive to moisture and air, as well as polar solvents, anion exchange processes, and thermal heating effects [9,11,12,13,14]. The intrinsic instability of CsPbX_3_ hinders its further development and future applicability in the field of optoelectronics [15,16]. The surface states cause poor photoelectric efficiency in perovskite-type devices, which are typically caused by surface bond recoil and surface contamination [17]. Therefore, it is crucial to develop an effective approach to enhance its stability. Consequently, numerous protection techniques, including surface passivation, have been developed. Protection approaches are an essential research topic for many real applications because they have a major influence on the stability and several physical features of perovskite-type materials, such as improved optical responses, surface changes, and surface passivation states [18,19]. Surface passivation techniques have important effects on optical properties by preventing surface states and surface defects and suppressing emission at depth. The presence of surface states has a stronger effect on the emission of shorter wavelengths than on the emission of longer wavelengths and can lead to negative thermal cooling behavior [20]. On the other hand, surface defects have a significant negative impact on the optical and electrical efficiency of the device. Therefore, the surface passivation of perovskite can improve both the PL stability and prevent halide segregation. For this purpose, these techniques may include encapsulation or surface radical polymerization by organic polymer matrices [15,16,21,22] or by mesoporous inorganic dielectric compounds, such as SiO_2_ [23,24,25,26,27,28,29,30] and TiO_2_ [31,32,33,34].

Haifeng Gao et al. [15] described photoinduced surface radical polymerization from CsPbBr_3_ perovskite nanocrystals (PNCs) and prepared PNC/polymer core-shell nanoparticles to improve the perovskite stability in polar solvents. Surface polymerization did not affect the lattice structure of CsPbBr_3_, but introduced surface-tethered PMA chains with controlled radical polymerization properties. Another group used CsPbBr_3_ perovskite NCs for photocatalyst PET-RAFT polymerization [16].

Appropriate processing conditions are required in encapsulation materials to ensure high optical and mechanical quality preforms, as well as the polymer’s compatibility with the perovskite and solvents. Also, it should be transparent and non-crystalline to certify that optical features are conserved. Furthermore, the refractive indices of the polymer should differ significantly from those in the perovskite layer for the periodic reflector to have a large bandwidth [35,36]. Due to the mechanical and chemical qualities necessary for encapsulation materials, PMMA has been widely employed as a material of choice in optical fiber production based-plastic [35,37], microfluidics technology [38], and optoelectronic applications [39]. PMMA’s optical characteristics are regulated by the introduction of noble metal nanoparticles [40], whereas the metallization of PMMA is employed for electrophoresis [39,41] and di-electrophoresis on microfabricated devices [42,43].

As a result, this study analyzes a PMMA selection that meets all of the following optical property requirements. Due to its lower crystallinity than homopolymers and hence better solubility, PMMA is employed as a light-emitting encapsulating material for PQDs [17]. The effects of the surface passivation layer formed by PMMA polymer coatings on the top surface of CsPb(Br_0.5_Cl_0.5_)_3_ and its thickness effect on the optical studies were investigated in our earlier study [2]. By comparing the properties of different PMMA polymer thicknesses to bare perovskite films, the ASE property analysis demonstrated the role of different PMMA polymer thicknesses. Moreover, the evolution of PL in perovskite materials with increasing pump energy density shows the presence of two regimes, and the origin of the two peaks has been investigated in the literature [44,45,46,47]. Thus, the transition from spontaneous emission (SE) to ASE was predicted. As mentioned earlier, PL emits broad emission spectra below the threshold energy; however, above the ASE threshold, this broad will be divided to form a smaller peak characterizing the ASE state [44,45,46,47]. In some materials, both stimulated and spontaneous emissions are caused by two forms of excitations, namely single excitons and double excitons (bi-excitons) [48,49]. Due to their ability to produce coherent quantum state combinations, bi-excitons are essential for quantum information [49,50,51,52,53,54,55]. The bi-exciton is produced by the binding of two-photon absorption or two excitons in condensed exciton systems. It can also be generated by excitation from the single exciton to the bi-exciton state [52]. Xing et al. [51] proposed the occurrence of an ASE state on the lower energy side of the exciton band, even below the Mott transition limit, based on the extensive studies of Ding and coworkers [56] on (Zn, Cd)Se/ZnSe quantum well lasers. Returning to the determination of the emission type in the two ASE regimes, in the spontaneous emission regime (below the ASE threshold), exciton emission dominates over bi-exciton emission, which can be attributed to non-radiative decay acceleration. In low-dimensional structures, bi-exciton emission is a fast decay (short lifetime) and is limited by non-radiative recombination. The fast decay of the bi-exciton emitter is related to the cooling of the hot excitons toward the state of the band-edge. Auger recombination (non-radiative) is dominant at high fluences because the extra energy generated by electron-hole recombination is transferred to electrons or holes, which are then excited to higher states within the same band rather than producing photons (radiation process) [51,52,53,54,57,58]. Therefore, bi-exciton emission is not noticeable at low fluence in the spontaneous emission state. This implies that the recombination duration at exciton and bi-exciton emission energies, as well as the estimation of Auger recombination losses, can be used as an approach to determine whether rapid recombination in perovskites originates from the bi-excitons. On this basis, the presence of the bi-exciton state is useful for studying carrier-energy relaxation in perovskite materials, as well as that in typical semiconductor QDs. The similarity between the perovskite materials and this type of semiconductor (such as PbSe or CdSe) represented in the optical gain could result only from the bi-exciton/exciton transition produced by the double degeneracy of the electronic states [52]. Compared with typical semiconductor QDs, perovskites have a very short recombination lifetime [52,54]. In perovskite materials, a significant inhomogeneous broadening criterion relative to the homogeneous thermal broadening criterion is not met [56,59,60,61].

According to Priante et al., surface states and bulk defect recombinations produce two ASE peaks. The first one appeared at a higher energy, with a limited density of states (DOS), and the second one appeared at a lower energy with a much higher DOS [46]. Kondo et al. attributed the formation of a low-energy peak to bi-excitons [44]. Chan et al. showed that surface treatment of CsPbBr_3_ nanoparticles not only increased their PLQY and extended their stability in the air from several days to several months, but also increased their trion PL lifetime [62].

Surface defects can also be used to explain the different emission types, e.g., the limited DOS of the higher energy peak versus the much larger DOS of the lower energy peak. As the excitation energy increases, the photogenerated excess carriers begin to fill the accessible states. As a result, this work focuses primarily on the vapor deposition of successive perovskite and PMMA films, which is one of the best fabrication techniques for smooth and hole-free films. In optoelectronic devices, the combination of thermal vapor deposition and surface passivation results in good ASE features (low threshold and high photostability) and increases the active layer’s light amplification efficiency.

The emission of bi-excitons is fundamentally a nonlinear phenomenon, so a high excitation fluence and/or a low temperature (e.g., 4 K) are generally required to observe them. Therefore, in the ASE spectrum, the link between high pump power density and/or low temperature and surface passivation is very important for the emergence of these emission species in the two ASE regimes [63,64]. Due to their lower self-absorption compared with conventional excitonic lasers, bi-excitons are also important for lasing. Therefore, new materials for bi-exciton emission with low excitation energy/power density are required for optoelectronic applications related to bi-excitons. In addition, bi-exciton emission is generally studied in low-dimensional structures. Thus, the fundamental goal of this work is to study and analyze the absorption coefficient, binding energy, and ASE properties of CsPb(Br/Cl)_3_ bulk as a function of surface passivation. The investigation will then focus on the ASE emission type with extensive research being conducted on the dependence of the bi-exciton emission ASE and surface passivation. Finally, the stability of the bi-exciton emission will be investigated.

## 2. Materials and Methods

### 2.1. Materials

The perovskite precursors: Lead chloride (PbCl_2_), lead bromide (PbBr_2_), cesium chloride (CsCl), and cesium bromide (CsBr), were provided from Sigma-Aldrich (St Louis, MO, USA). A polymethyl methacrylate (PMMA) polymer with the chemical formula (C_5_O_2_H_8_)n and molecular weight (Mw) equal to 120,000 g/mol was also purchased from Sigma-Aldrich.

### 2.2. Synthesis of CsPb(Br/Cl)_3_ Perovskite and PMMA Polymer Films

The synthesis process is described in detail [2]. Briefly, CsPb(Br/Cl)_3_ and [PMMA/CsPb(Br/Cl)_3_] films were created on the glass substrate by TET from a mixture of the main components of the perovskite precursors of CsX and PbX_2_, where X = Br/Cl in an equal stoichiometric ratio. In a single boat, the precursor components were charged in sequence. The PMMA layer was then deposited above the perovskite film by TET to also encapsulate the perovskite surface. The appropriate amount of PMMA powder loaded into the boat to achieve the desired thickness was then adjusted to 400 nm with an accuracy of less than 10%. Finally, the encapsulated and bare films were annealed for 15 min at 120 °C.

### 2.3. Structural Characterization

Scanning electron microscopy (SEM) (JEOL-7600F, JEOL, Tokyo, Japan) was used to examine the film morphology, and X-ray diffraction (XRD) was used to investigate the crystal structure and determined the phases of the material. Here, an XRD system (Miniflex 600, Rigaku, Tokyo, Japan) with copper K_α_ radiation (λ = 1.5418 A) was used. The scan angle (2θ) range was taken from 10° to 60° and the scan rate (a step size) was adjusted to 0.02° at 3°/min.

### 2.4. Optical Characterization

First, the encapsulated and bare films’ absorption spectra were measured with a UV-Vis spectrophotometer (V-670, JASCO, Tokyo, Japan). Then the PL spectra of the same samples were recorded using a fluorescence spectrophotometer (FP-8200, JASCO, Japan) in the 440–520 nm wavelength range. The films thicknesses measurements were taken by a Dektak (150 Stylus Profiler, Bruker, Billerica, MA, USA).

### 2.5. ASE Measurements and Data Analysis

The pumping energy-dependent PL and ASE intensity spectra were recorded for the encapsulated and bare samples. For sample excitation, Q-switched of third-harmonic generation (THG) produced by Nd:YAG picosecond laser LOTUS II (LOTIS, Minsk, Belarus) (pulse duration: 70–80 ps; repetition rate: 15 Hz) was used. The laser beam was focused by a cylindrical lens (f = 10 cm) after passing through a hall with a 2 mm diameter aperture. The signals emitted from the sample’s edges and near the ends of the excitation strips were routed through an optical fiber. ASE signals were collected using an Ocean Optics QE65 Pro spectrograph (Ocean Optics, Orlando, FL, USA). The pumping energy density of the laser signal was attenuated using laser pumping to explore the PL and ASE states. A thermal sensor head (LM-P-209, coherent) was used to monitor the energy of the laser pumping.

### 2.6. Time-Resolved Photoluminescence Measurements (TRPL)

TRPL measurements were achieved from the sample after being excited by the above laser. Special filters were employed to choose the emitted light from the sample. A lens was also employed to concentrate and guide the emitted light to a fast and sensitive photodiode (APD110). To evaluate the temporal sequences, this photodiode was attached to a fast real-time digital oscilloscope with a two-channel (Tektronix TDS 380, bandwidth 400 MHz, sampling rate 2GS/s).

All measurements that were taken for the samples were carried out at room temperature (295 K).

## 3. Results and Discussion

The results include the analysis of CsPb(Br/Cl)_3_ and PMMA/[CsPb(Br/Cl)_3_] configuration films on a glass substrate. The CsPb(Br/Cl)_3_ and PMMA/[CsPb(Br/Cl)_3_] configurations are coded as follows: bare and encapsulated samples, respectively. The thicknesses of the CsPb(Br/Cl)_3_ film and PMMA layer were set to 400 nm for both layers, with errors of less than 10%.

### 3.1. Structural and Morphological Properties

**Figure 1:**
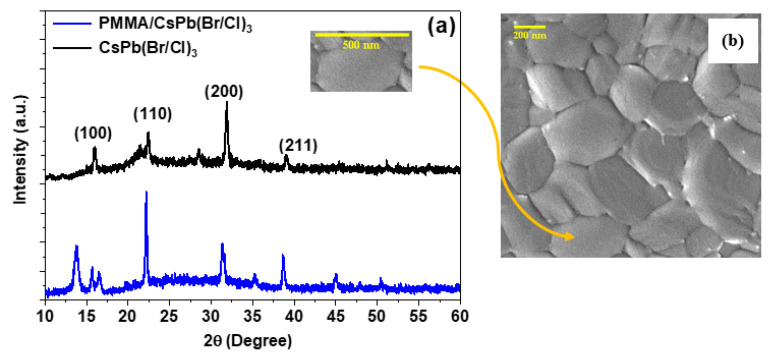
(**a**) XRD patterns (**b**) SEM picture of CsPb(Br/Cl)_3_ perovskite thin films deposited in microscopic glass (scale bar, 200 nm).

Figure 1a shows the X-ray diffraction (XRD) peaks and the analysis of the bare and encapsulation perovskite film. As shown in Figure 1a, the XRD pattern indicates that the mixed CsPb(Br/Cl)_3_ perovskite is crystalline and has several diffraction peaks, especially angular peak positions (2θ) at 15.98°, 22.36°, 31.84°, and 38.98°, corresponded to the (100), (110), (200), and (211) crystal plane diffractions. These can be indexed to the CsPbBr_3_ phase of (JCPDS Card No. 72-7929) [8,65] with a peak shifting to a higher angle (2θ), rather than that corresponding to the pure sample, with introduced CsPbCl_3_ (JCPDS Card No. 04-024-6243) [8,66]. The peak at 22.36° is quite broad and has a shoulder peak at 21.43° that may be considered to result from surface states due to those disappeared later in the encapsulation sample.

The peak positions indicate the formation of an orthorhombic phase in the Pnma space group, which is consistent with previous work [10,67,68,69,70,71,72,73,74,75], and this phase is expected since both CsPbCl_3_ and CsPbBr_3_ perovskite exhibit an orthorhombic crystal structure in the RT and bulk structure. The presence of the (200) and (100) planes in the role of a plane and the secondary plane suggests the presence of a high crystalline, high-purity phase free from the defects from perovskite primary materials [17]. The XRD pattern of the encapsulation film revealed the same peaks without any sharp diffraction peaks, confirming that PMMA is amorphous (non-crystalline nature), except for the peak at 2θ ≈ 13.70°, which reflects the ordered packing of polymer chains, and the second peak at 16.52°, which can denote the ordering inside the main chains with their intensity decreasing systematically. This explains why PMMA thin films are homogeneous [76]. Additionally, the peak at 21.43° dispersed after surface passivation

An SEM picture was used to examine the morphology of both samples. As shown in Figure 1b, the image SEM shows the morphology of a bare sample deposited on a substrate using a TET. The coverage of the substrate with bare CsPb(Br/Cl)_3_ perovskite material is quite uniform throughout the substrate, with crystal domains consisting of grains of about 0.346 μm in size. The mixed halide of Cl and Br affects the domain size of the perovskite. The high-quality deposition method here also has a significant effect on the highly crystalline hole-free structure. For the encapsulated sample, the SEM image, however, could not be recorded because PMMA is sensitive to electron beams of the SEM, resulting in damage to the PMMA trenches, which is consistent with prior reports [2,77].

### 3.2. Optical Characterization

#### Measurements of UV-Vis Absorption & Steady-State Photoluminescence

**Figure 2:**
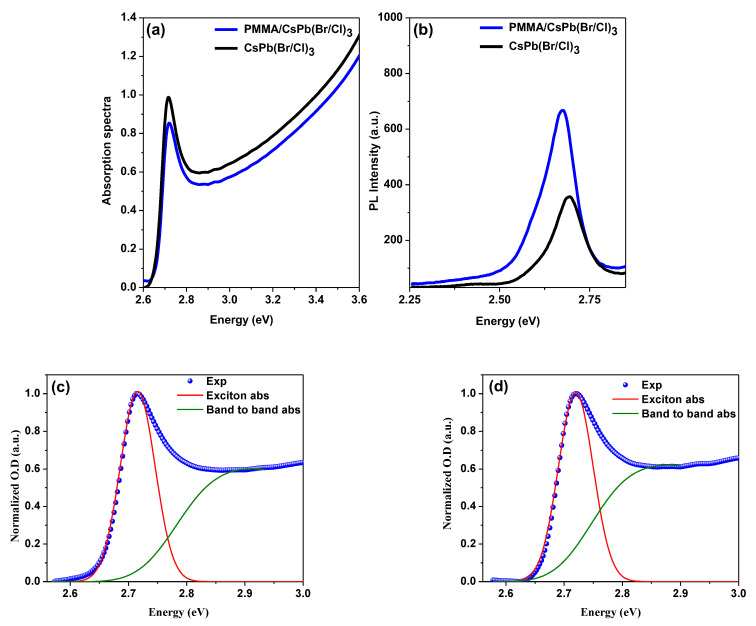
(**a**) Optical absorption and (**b**) emission; spectra of bare and encapsulated samples. Excitonic and band-to-band absorption for (**c**) bare and (**d**) encapsulated samples, with experimental data.

The optical absorption spectra in the UV-Vis region of the bare and the encapsulated samples in the thin film state, measured at RT, are shown in Figure 2a. The absorbance spectrum decreases with rising wavelength, with an absorption edge at ~2.77 eV (447 nm) and a sharp peak at ~2.73 eV (455 nm). This peak shows the presence of an excitonic transition, as well as the film’s high crystal quality [78,79]. The absorption onset can be seen near the band edge of the pure CsPb(Br/Cl)_3_ perovskite [2]. As for the peak absorption position of the films, it’s clear that the absorption spectrum exhibits three broad features: band-to-band transitions, a strong exciton peak, and a low energy absorption tail below the band gap [2,80,81]. The matching of the absorption spectra of both samples confirmed the crystal structure had not changed. The PL spectra for both configurations, measured at RT with an excitation wavelength of the incident light beam of λ_ex_ = 355 nm, are shown in Figure 2b. The PL peak position of the bare sample, which is located between CsPbCl_3_ and CsPbBr_3_, shows that the band structure of CsPb(Br/Cl)_3_ halide solid solutions can be controlled by the exchange of halogen ions. Furthermore, the peak position of the PL band coincides with the peak position of the exciton absorption band. As a result, it is possible to infer that the PL from both films corresponds to the emission of free excitons [82].

The PL spectrum, as shown in this figure, has sharp PL peaks at about 2.675 eV and 2.693 nm, with the half-width (FWHM) measured to be 97.00 meV and 93.72 meV for the bare and encapsulated samples, respectively. The introduction of the PMMA polymer layer resulted in a slight shift in the peak position and a decrease in PL FWHM, which can be attributed to the film enhancement, followed by an improvement in the PL sign due to the PMMA layer, as shown by the PL peak position and FWHM results. Some of the optical characteristics derived from the absorption and PL spectra are listed in Table 1. The encapsulated sample exhibits variations in both spectra intensities, with absorption intensity decreasing and PL intensity increasing significantly. The surface passivation is responsible for the considerably higher PLQYs of the encapsulated samples compared to the bare samples. This increase suggests that the number of non-radiative recombination sites on the surface decreases as a result of effective passivation of surface trap states, leading to a decrease in deep traps in the density of states measurement. Based on the perovskite thin films developed in this research, it can be assumed that surface defect passivation aids in the achievement of bi-excitons ASE. The positions of the absorption edge and the PL peak corroborate a blue shift in PL, as well as a reduced Stokes shift and increased PLQY with the passivated surface. [17]. Film scattering increases with PMMA film, which is visible in the PL spectra (Figure 2b). The PL measurements do not reflect the optimal film thickness because absorption in the surface layer above the perovskite layer can be reduced with higher pulse energies (laser), as discussed in section ASE.

E_g_ was calculated by calculating the Summerfield factor S(E), where E indicates photon energy. The films’ absorption coefficient α(E) was calculated using an equation that considers the S(E) [80]. For further explanation and clarification, the corresponding equations have been included in previous work [2,8,83], and the data are shown in Figure 2c,d and are tabulated below.

### 3.3. ASE Pump Fluence-Dependent Studies

It’s known when a material is pumped to induce a population inversion, the ASE process is a critical and fundamental step in evaluating its suitability for amplification. Optical cavity feedback is required to create a laser that operates at a laser threshold. A cavity-free configuration (ASE) is accomplished here because the ASE provides a useful foundation for determining a material’s suitability for gain applications. Although ASE is much more intense, the laser has a much higher degree of coherence than ASE [34,55].

As mentioned in the introduction section, it is widely believed that the use of nanostructure is the only way to achieve efficient light emission from CsPbX_3_ perovskites. High-quality films were synthesized from perovskite bulk using a high-development method (thermal evaporation), and a picosecond laser was used as an excitation pumping source (λ_ex_ = 355 nm). The impact of surface passivation by the PMMA polymer coating was then investigated, and its properties and optical behavior were compared to that of the bulk phase bare films. So, to achieve the research goal, namely the occurrence of bi-excitons ASE, the first step was to optimize the thicknesses of polymer on the CsPb(Br/Cl)_3_ thin films. PMMA is a non-crystalline polymer with a transparent structure. Moreover, the refractive indices of a polymer at the wavelength used for perovskite emission are different from those of perovskite, which allows for a wide bandwidth. Additionally, bi-excitons are more likely to form at high excitation levels [84]. Due to their properties of producing coherent combinations of quantum states, bi-excitons are important for quantum information [49,50,51,52,53,54,55]. Since the emission of bi-excitons is a nonlinear process, a high excitation energy density or/and a low temperature are usually required to generate a bi-exciton [63,64]. Optoelectronic applications related to bi-excitons will benefit from materials that emit bi-excitons at low excitation energy density. Surface defects can also be used to explain the emission modes, such as the limited DOS of the higher energy peak versus the much larger DOS of the lower energy peak.

**Figure 3:**
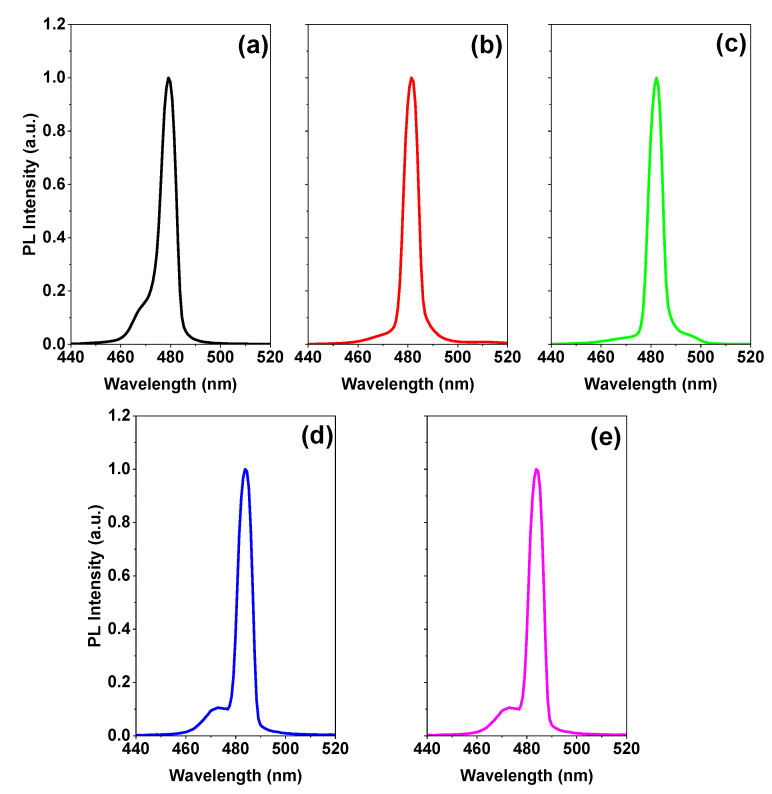
PL of CsPb(Br/Cl)_3_ thin films above the energy density threshold for different thicknesses of a passivation layer on; (**a**) without polymer (0 nm), (**b**) 100 nm, (**c**) 200 nm, (**d**) 400, and (**e**) 500 nm.

Figure 3 shows the PL spectrum above the ASE threshold for PMMA polymer thicknesses of 0, 100, 200, 400, and 500 nm. Bi-excitonic ASE appears for a thickness of 400 nm and above, which is why we chose the current thickness (400 nm) at which the rest of the study will be completed for its thickness. After depositing PMMA with a thickness of 100 or 200 nm, the disappearance of the peak located around 468 nm is attributed to spontaneous emission which means that the ASE became dominant in these films owing to the lowering of the ASE threshold [2].

For more information, the emission spectra of the encapsulated samples are investigated below, along with the ASEs properties and a comparison of the ASE performance of the encapsulated sample surfaces with the bare surface films.

**Figure 4:**
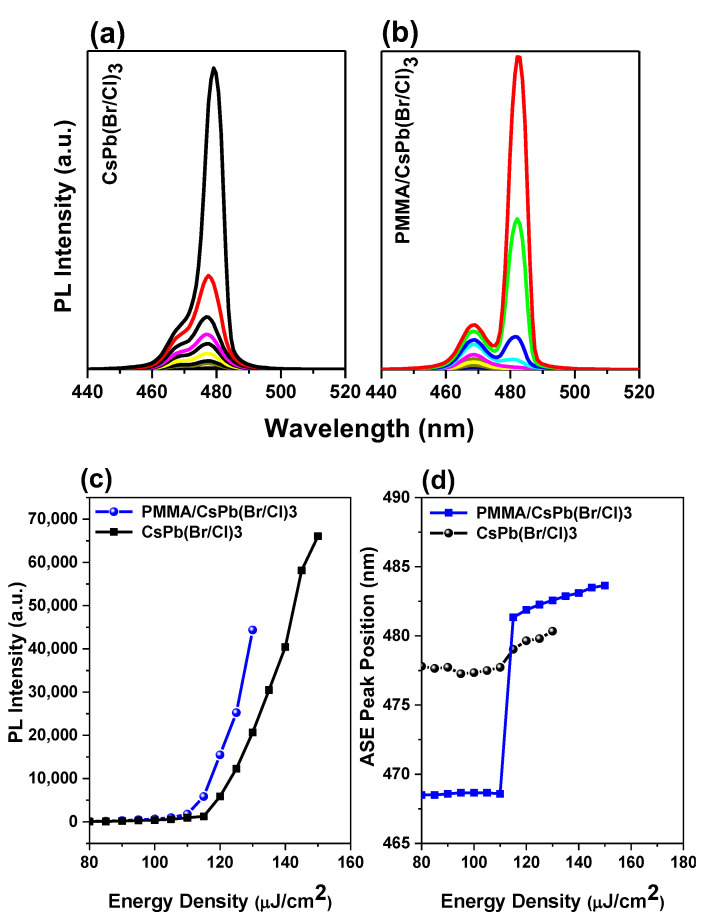
ASE Pump-fluence relationship for CsPb(Br/Cl)_3_ perovskites thin film: (**a**) bare sample, and (**b**) encapsulated sample. (**c**) The PL intensity and (**d**) peak position vs their energy density of CsPb(Br/Cl)_3_ thin films, both bare and encapsulated samples.

The ASEs were formed by increasing the pumping fluence in the bare and encapsulated samples, as shown in Figure 4a,b. At low energy, the spectrum is wide and featureless. While the energy exceeds the threshold, a smaller peak at redshift forms, showing the occurrence of an ASE state in both samples. The ASE pump energy density threshold was calculated by observing the simultaneous incidence of the emission spectrum narrowing and the ASE peaking in the low energy region of spontaneous emission, followed by fast evolution. Another method for determining the threshold is to observe the intensity, where a slope fracturing will cause a nonlinear growth in PL intensity in a curve between output and input intensity. Figure 4c shows a rapid increase in peak intensity versus energy density near the threshold, indicating the transition from spontaneous emission to stimulated emission. The PL linewidth decreased with increasing pump energy density for all samples, confirming the occurrence of an ASE state. Above the threshold of ASE, the FWHM ASE was saturated. The ASE formed at 473 nm in bare films was shifted to 480 nm in the bare sample. For the encapsulated sample, the ASE was generated at 474 nm and shifted to 482 nm. The sudden wavelength shift with increasing pump fluence was also followed by an immediate change in PL intensity that coincided with a drop in FWHM. The onset of the stimulated emission is accompanied by an abrupt rise in ASE strength and a narrowing of the spectrum across the threshold pump area. (FWHM 6 nm). The ASE peak (7 nm) was discovered to be red-shifted in comparison to the PL peak.

The PL spectra contain two peaks that help determine the FWHM, which is difficult to do using the conventional calculation method. Thus, the Peak-Fit tool was needed to fit the massive amount of data. The data and Gaussian fit of dual peaks were analyzed by the Peak-Fit with a custom Python-based program developed by our research team.

Since the variation in the slope occurs while the PL spectrum is already narrow, the thresholds for the change in the slope are slightly higher than those determined by the FWHM drop. Therefore, the threshold is calculated by averaging the minimum energy density so that the ASE regime can be evaluated at five different locations in the sample.

The thresholds were predicted to be 115 µJ/cm^2^ and 110 µJ/cm^2^ for bare and encapsulated film, respectively, as shown. Initially, the slower PL relaxation and the higher intensity were attributed to the defect’s passivation on the film surface, as well as approximately 5% of the ASE threshold lowering. Surface smoothing reduces incident pump light loss at the interface between the air and perovskite thin film, resulting in greater emissivity and more powerful light emission, potentially improving ASE performance. This performance will lead to enhanced thin films for laser applications and other photonic devices with active layers.

The laser peaks at 474 nm moved to 482 nm, and the position of the ASE peak’ shifted as a function of pump energy density (Figure 4d). There are few reports on the formation origin of these two peaks in perovskite-type materials [44,46,47,51]. Many factors, including reabsorption effects during single exciton amplification, [85,86,87] biexciton recombination, [52,88,89,90,91,92], temperature effects due to the pump source heating the sample, defect transitions, and bandgap renormalization [52,93], may have contributed to the ASE redshifts [52,85,86,87,88,89,90,91,92,93,94,95]. The two peaks are caused by surface defects, according to Priante et al., with the lower energy peak having a much higher DOS and the greater energy peak having a limited DOS [46]. When the excitation energy is raised, there is no defect-like signature for the wide peak, which has a limited DOS, and the photogenerated excess carriers begin to saturate the PL intensities.

This data supports the hypothesis that the PL peak is caused by bulk defects. As a result, the PL spectra contained a single narrow peak and a broad peak, which were linked with surface states and negligible bulk defect recombination [79]. This means these samples are good source materials for light emitters. This idea was prompted by the fact that high-quality single crystals had just one peak [96], whereas polycrystalline samples grown under very mild circumstances contain two peaks [46], which inspired this hypothesis. Kai Chen et al. [47] studied for the absorption and ultrafast broadband PL lead to discover a short-lived PL emission (10 ps) produced by uncorrelated hot charge carriers above the band gap. After the thermalization of the heated carriers, the ASE peak grows on the lower energy side in a stronger and sharper form [47]. Different fabrication methods of the sample can cause very different behaviors and the origins of the two peaks are varying. The self-absorption effect is predicted for the ASE based on the reabsorption effect observed when the absorption band edge coincided with the PL emission at lower pump energy (spontaneous emission region). Due to the sensitivity of ASE to optical loss, the reabsorption effect results in a redshift of the peak wavelength of PL [85,86,87], which is even more pronounced. The more intense the PL reabsorption is when considering the propagation of PL through the PQDs film, the thicker the film is. Bi-exciton emission reduces the effects of self-absorption compared to conventional exciton emission.

### 3.4. Bi-Excitonic ASE Formation and Stability Studies

Neutral excitons, trions, dark excitons, bi-excitons, defect-bound excitons, or excitons in the localized state can be localized by studying the power-dependent PL radiation and decomposing the spectrum into individual Lorentz peaks [84]. In general, excitons and bi-excitons, two types of excitations (luminescent quasiparticles), are responsible for both triggered and spontaneous emissions in some materials [48,49]. Two excitons can be bound together in concentrated exciton systems, two photons can be absorbed, or the single exciton state can be excited to the bi-exciton state. A bi-exciton consists of two neutrally bound excitons that are redshifted due to Coulomb processes, unlike unbound excitons [52]. Two electrons and two holes form bi-excitons, which have been observed to split into two excitons, release two photons in sequence, or produce a pair of entangled photons.

**Figure 5:**
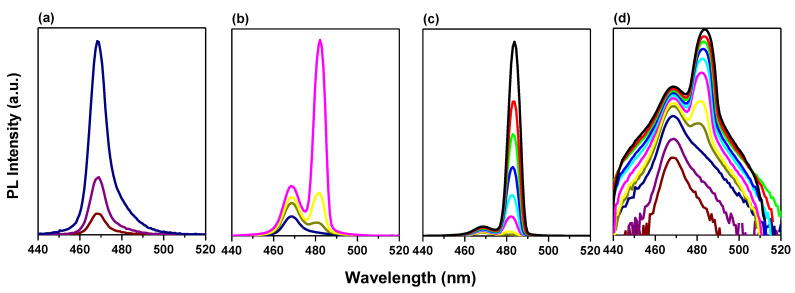
PL of (**a**) single-exciton emission, (**b**) develops bi-exciton emission, (**c**) emission at even lower and higher fluences, and (**d**) logarithmic scale of the d spectra.

In particular, for an entrapped sample, two emission peaks are detected at RT. To determine the causes of these two emission peaks, we performed excitation energy-dependent studies PL (Figure 4a,b). As the excitation energy increases, both emission peaks become stronger. (Figure 5a,b). Free excitons (denoted as X) and bi-exciton emission are two categories for the two emission peaks (denoted as XX). The bi-exciton emission peak can be observed at excitation energy as low as 3.52 mJ at RT for an encapsulated sample covered by PMMA with a thickness of 400 nm, which corresponds to an energy density of 110 µJ/cm^2^. Due to the high exciton density in dense samples, we also investigated the thickness-dependent emission of bi-excitons (Figure 3).

The single-exciton PL (low-intensity) develops firstly into bi-exciton emission, followed by gain narrowing when emission transformed from spontaneous to stimulated due to higher fluences (Figure 5). The pump energy-dependent analysis PL shows that the emission contribution of bi-excitons increases sharply with energy density. Here, higher pump energy and better light capture together lead to high excitation (about twice as much). The fact that the bare film does not appear to have the same dependence on pump energy suggests that PMMA-modified films have higher excitation due to light trapping. The modified film appears to have higher PL contributions from defect-bound and localized excitons due to defect accumulation in addition to an increase in bi-exciton emission. However, the overall relationship between emission intensity and excitation remains linear within the range of excitation fluences studied.

**Figure 6:**
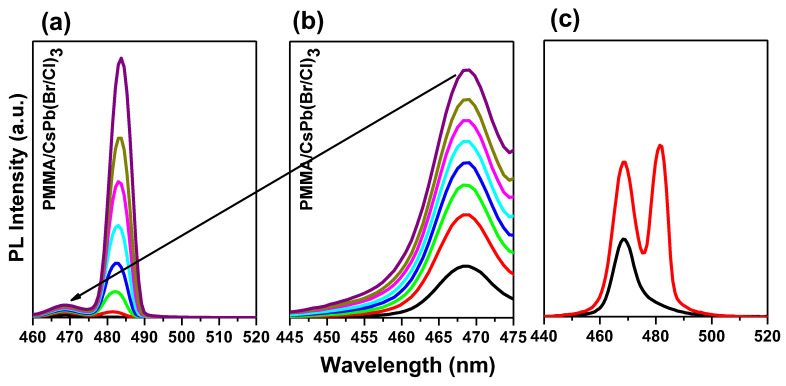
ASE Pump-fluence relationship for encapsulated samples configuration thin film: (**a**) all peaks, (**b**) magnification of low energy peaks, and (**c**) tow spectrum before and after threshold.

**Figure 7:**
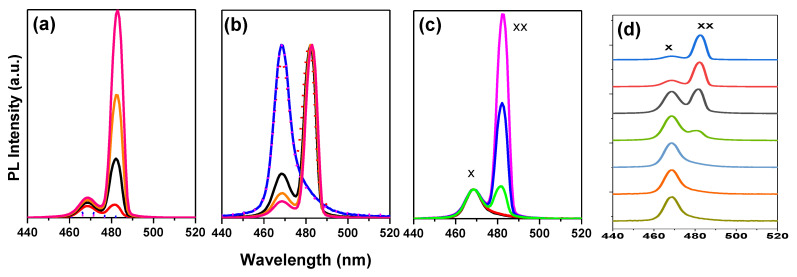
(**a**) Evolution of PL spectra with increasing optical pump fluence for the encapsulated sample. Normalized PL X and XX bands’ excitation intensity dependences concerning (**b**) XX band and (**c**) X band, and (**d**) The peak wavelength shifted from approximately 470 nm (BE_1_) to approximately 480 nm (BE_2_).

At low pump fluence, only the free exciton PL at 2.616 eV and some bound exciton bands at 2.573 eV are detectable; however, at higher pump fluence, the spectra are dominated by a new PL band located at 43.42 meV below the free exciton PL. This new PL band, which is labeled XX in Figure 6c, is seen at a modest pump fluence of 110 µJ/cm^2^ and evolves rapidly with pump fluence. Typically, a shift from ASE relative to the spontaneous PL is the main indicator that the bi-exciton/exciton transition is the process underlying ASE. The bi-excitonic ASE band often occurs at energies (E_XX_) lower than the energy of the single-exciton emission E_X_, which is due to the interactions between straight Coulomb bands. The energy difference E_bXX_ = E_X_ − E_XX_ accounts for the binding energy of the bi-excitons. At the ASE threshold in CsPb(Br/Cl)_3_ layers, the energy difference between the peak energies PL (spontaneous emission) and ASE is less than about 43.42 meV, which is consistent with values from other studies. As mentioned above, regarding fitting the absorption model to the spectrum, an exciton binding energy E_bx_ was determined to be 70.20 meV. A higher exciton binding energy increases the probability of bi-exciton formation [89]. The energy difference between the peak positions of exciton and bi-exciton yields a bi-exciton binding energy of about E_bxx_  =  26.78 meV, making bi-exciton and exciton have a ratio of 38.15%. So, these values show that the encapsulation layer possesses large exciton and bi-exciton binding energies of 70.20 meV and 26.78 meV, respectively, giving rise to stimulated emission from bi-excitons at room temperature. Based on Figure 7, the bi-exciton binding energies correspond to 38.15% of the estimated 70.20 meV excitons binding energy (Figure 2d).

**Figure 8:**
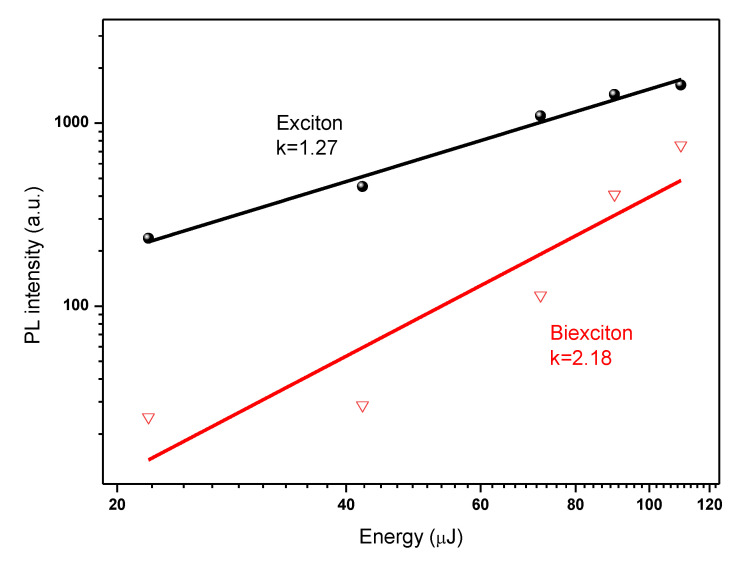
PL intensity vs. excitation energy. The XX intensity obeys a power-law function of the excitation power (X, K = 1.27 and XX, K = 2.18).

A quadratic dependence of the intensity of the ASE integral on the excitation density is considered by many to be additional evidence for the bi-exciton structure of the ASE band. Based on generally accepted arguments in the literature, our experimental data are therefore consistent with the widely held assumption that the ASE band has a bi-excitonic origin [54]. The integrated emission intensity is plotted against excitation energy in Figure 8 using the data from Figure 7a. Based on the results of the fit, the two emission peaks can be categorized as free exciton (denoted as X) and bi-exciton emission (denoted as XX) (Figure 8). The energy-dependent integrated emission intensity can be well described by I_PL_ I_ex_^k^, where I_PL_ stands for the integrated PL intensity, I_ex_ stands for the excitation power, and k stands for the fitting parameter [2,34]. The k values for these two emission maxima are 1.27 and 2.18, respectively, (Figure 8). The integrated intensities confirm that the redshifted peak is due to bi-exciton emission. The amplitudes I_x_ and I_xx_, respectively, exhibit linear ($I\propto I_{PL}$) and quadratic ($I\propto I_{PL}{}^{2.18}$) dependences on the excitation fluence. The latter confirms that a bi-exciton transition is what is causing the red band (Figure 8) and intensity-dependent spectra are caused by a bi-exciton transition because the stimulated emission originates at the energy position of the bi-exciton transition. The integrated stimulated emission intensity exhibits unambiguous threshold behavior, with the biexciton PL transitioning to stimulated emission at an ultralow threshold of 110 µJ/cm^−2^.

The PL levels of the X and XX bands are shown in Figure 7 as a function of the fluence of the excitation pump. The intensity of the X band PL increases with the square root of the excitation intensity above 110 µJ/cm^2^ and is proportional to the intensity of the excitation pump fluence below this threshold. On the other hand, the XX PL cence band increases in proportion to the excitation intensity up to 110 µJ/cm^2^ before continuing to increase with the square of the excitation intensity. Thus, in the measured intensity range, the XX band intensity is proportional to the square of the X-band. The dependences of the X and XX bands on excitation intensity are very comparable to those of exciton and bi-exciton recombination luminescence [89,97]. This proves that excitons are superior to bi-excitons, while their role in ASE release is still recognized. The origin of PL’ in bi-exciton recombination may serve as an explanation for why there is a particularly striking superlinear increase in the integrated PL of the narrow band with intensity.

**Figure 9:**
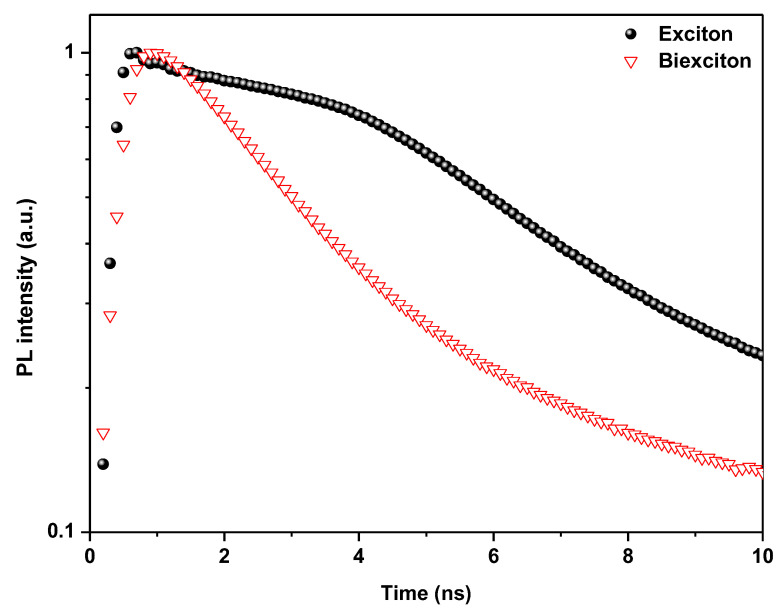
TRPL intensity (logarithmic scale) of the X and XX emission after excitation at time zero and for 100 and 120 µJ/cm^2^ excitation fluence for black and red lines, respectively.

Figure 9 shows the time-resolved PL spectra of the sample in both excitation regimes. Fitting a multi-exponential function to the spectrally integrated exciton decay results in an effective lifetime of τ_x_ = 7.41 ns as well as a lower intensity tail and a decay constant of 55 ns.

At higher excitation fluence, the PL trace has a slightly faster decay time of τ_2_ = 4.39 ns, with a 33 ns tail. We can estimate a biexciton lifetime of τ_xx_ = (τ_2_ − I_x_τ_x_)/I_xx_ = 1.10 ns by focusing on the picosecond decay times and using the respective ranges of exciton and biexciton emission from Figure 5 at the same excitation fluence. The τ_xx_/τ_x_ ratio of 1:6.7 rules out the possibility that nonradiative Auger recombination dominates the biexciton lifetime. In a recent study, Auger lifetimes of up to 10 ns were found (see ref. [98]), which is almost an order of magnitude longer than the measured radiative lifetimes [89].

**Figure 10:**
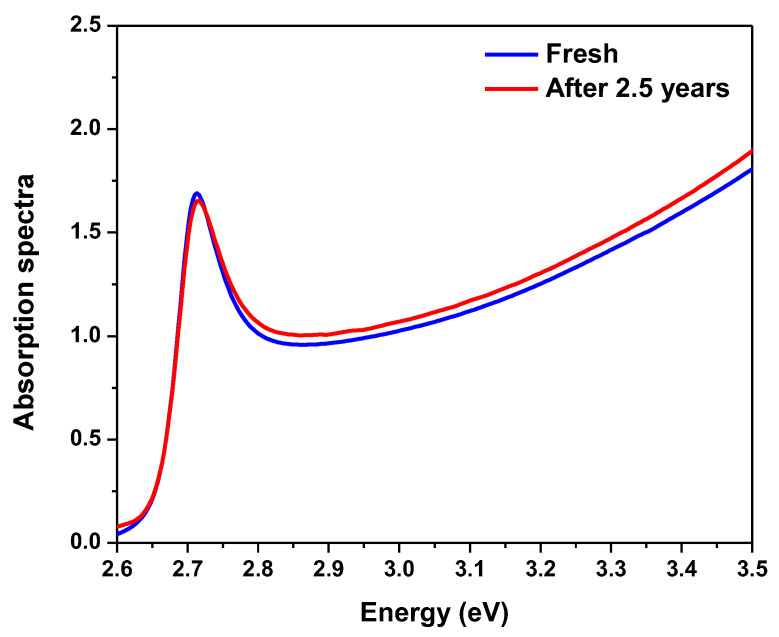
Optical absorption for encapsulated sample configuration thin film, fresh sample and after 2.5 years.

**Figure 11:**
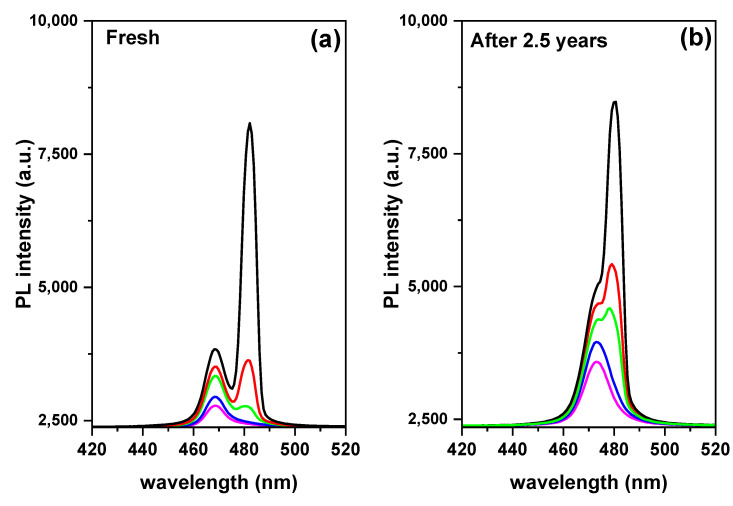
ASE Pump-fluence relationship for encapsulated sample configuration thin film to characteristics of bi-exciton stability, (**a**) fresh sample and (**b**) after 2.5 years.

Finally, the bi-exciton emission stability can be determined from the binding energy value. Figure 10 shows the optical absorption of the encapsulated samples configuration thin film in two states; fresh and after 2.5 years. The excitonic behavior changed with time, indicating a change in the exciton binding energy, but this change is relatively small. As mentioned above, a higher exciton binding energy increases the probability of bi-exciton formation [89,97]. The binding energy of biexcitons implies a residual in-plane center-of-mass confinement, which is advantageous in our case because biexcitons are stable at room temperature. Due to the fact that if the binding energy is greater than the thermal energy, as demonstrated by k_B_T = 25.46 meV, where k_B_ and T are the Boltzmann constant and room temperature, respectively, bi-excitons form stably even at room temperature [89]. Moreover, bi-excitons are more stabilized by localization than single excitons. So, exciton contributions are less than bi-excitons with time (Figure 11). The presence of bi-exciton emission at such low excitation pump energy density can be attributed to the as-synthesized crystals’ high crystalline quality and surface encapsulation [46,62]. It should be mentioned that the application of protection materials is a function of both encapsulation and surface passivation in a single protection process, which can preserve optical properties and endow perovskite films with high stability due to synergetic effects.

Additionally, after two years of storage at RT, the time-dependent PL demonstrated the blue shift relative to the initial PL. The irreversible photo-oxidation of perovskite is what causes the blue shift and photo-bleaching of perovskite [99].

## 4. Conclusions

Utilizing TET with a single source, a PMMA polymer coating was used as a unique surface passivation technique to cover the top layer of the CsPb(Br/Cl)_3_ perovskite films. The properties of the perovskite films and their optical responses with the coating were then compared with those of the bare perovskite films. The surface passivation layer improves the optical response. Surface modification-dependent optical responses can suppress surface states and enhance surface quality without affecting optical or structural properties. In addition, the ASE improved, which is essential for improving the performance of optoelectronic devices. In addition, surface passivation helps us to discover the bi-exciton emission in the top encapsulating perovskite layer at RT, which is caused by a strong nonlinear effect and enhanced Coulomb interaction. Although the bi-exciton emission is produced under the required conditions of high excitation power density and low temperature, this work succeeded in producing it with a low excitation energy density of 110 µJ/cm^2^ and at RT. The reason for this is the fundamentally nonlinear nature of the bi-exciton emission because the polarization direction is more skewed toward the crystallographic plane. At 26.78 meV, the significant binding energy of the bi-excitons is equal to 38.15% of the binding energy of the excitons. (70.20 meV). The ASE produced by CsPb(Br/Cl)_3_ bulk structures shows that light emission from sources other than CsPbX_3_ perovskite nanoparticles is possible. Optoelectronic applications involving bi-excitons, and novel materials for bi-exciton emission with low excitation power density are required. Our findings contribute to understanding bi-excitons formation and its important implications for modern optoelectronics and bi-exciton laser technology because they have a lower self-absorption effect than traditional excitonic laser technology.

## Figures and Tables

**Table 1:** Contains information on the PL, exciton, and PL peak positions, exciton and PL-FWHMs, and the E_g_ optical bandgap of the bare and encapsulated samples.

Compound	E_g_ (eV)	E_b_ (meV)	Exciton		PL		Stock Shift (meV)
			Peak (eV)	FWHM (meV)	Peak (eV)	FWHM (nm)	
CsPb(Br/Cl)_3_	2.787	67.0	2.719	70.67	2.675	97.00	41
PMMA/[CsPb(Br/Cl)_3_]	2.786	70.2	2.716	70.67	2.693	93.72	26

## Data Availability

Not applicable.
